# The impact of the COVID-19 and mpox outbreaks on behaviours associated with HCV infection among MSM: results from the prerandomisation phase of a clinical trial

**DOI:** 10.1097/QAD.0000000000004265

**Published:** 2025-06-20

**Authors:** Kris Hage, Anders Boyd, Udi Davidovich, Ellen Generaal, Elske Hoornenborg, Paul Zantkuijl, Marc van der Valk, Dominique Verhagen, Janneke Stalenhoef, Jan den Hollander, Eliane Leyten, Tania Mudrikova, Hayette Rougier, Thibault Chiarabini, Marc-Antoine Valantin, Gilles Pialoux, Pauline Campa, Janke Schinkel, Karine Lacombe, Maria Prins

**Affiliations:** a Public Health Service of Amsterdam, Department of Infectious Diseases, Research and Prevention; b Amsterdam UMC location University of Amsterdam, Department of Infectious Diseases, Meibergdreef 9; c Amsterdam institute for Immunology and Infectious diseases, Infectious Diseases; d Stichting HIV Monitoring; e Department of Social Psychology, University of Amsterdam; f Soa Aids Nederland; g Department of Internal Medicine, Jan van Goyen Medical Centre; h OLVG, Department of Internal Medicine, Amsterdam; i Department of Internal Medicine, Maasstad Teaching Hospital, Rotterdam; j Department of Internal Medicine and Infectious Diseases, Haaglanden Medical Centre, The Hague; k Department of Internal Medicine, University Medical Centre Utrecht, Utrecht, The Netherlands; l IMEA, Institut de Médecine et d’Épidémiologie Appliquée; m Infectious Diseases Department, St Antoine Hospital; n Infectious Diseases Department, Pitié-Salpêtrière Hospital, Sorbonne University, APHP; o Pierre Louis Epidemiology and Public Health Institute (iPLESP), INSERM 1136; p Infectious Diseases Department, Tenon Hospital, Sorbonne University, APHP; q Maison Médicale Chemin Vert, Paris, France; r Amsterdam UMC, Department of Medical Microbiology and Infection Prevention, University of Amsterdam, the Netherlands.

**Keywords:** COVID-19 restrictions, hepatitis C virus, men who have sex with men, mpox, sexual and drug use behaviours

## Abstract

**Objective:**

To assess whether the COVID-19 and mpox outbreaks affected hepatitis C virus (HCV) related behaviours among men who have sex with men (MSM) with a cleared HCV infection.

**Design:**

Longitudinal analysis from the international ICECREAM trial (2021–2024).

**Methods:**

During the prerandomisation phase (i.e., without any intervention) individuals completed questionnaires on sexual and drug use behaviours and whether the COVID-19 (since start trial) or mpox (shortly after the mpox outbreak in 2022) outbreaks caused changes in these behaviours, all referring to the preceding 6 months. We used mixed-effects logistic regression to model changes in behaviours due to COVID-19 or mpox measures and mixed-effects linear regression to model the average HCV-MOSAIC risk score, as a proxy of HCV-associated risk behaviour, over calendar time.

**Results:**

220 MSM (*n* = 117 from the Netherlands, *n* = 103 from France) were included. Among 208 that completed the baseline questionnaire, 171 (82.2%) were MSM with HIV. The proportion of individuals reporting any impact of COVID-19 restrictions on risk behaviours, mainly lowering number of partners, decreased from 74.7% in September 2021 to 6.7% in September 2024 (*P* < 0.001) and reporting any impact of mpox from 41.9% in November 2022 to 6.0% in September 2024 (*P* = 0.001). The average HCV-MOSAIC risk score remained constant over time (*P* = 0.59) and was consistently ≥2.0, indicating high reinfection susceptibility.

**Conclusion:**

HCV-related behaviours decreased when COVID-19 and mpox measures were in place. However, individuals still engaged in behaviours associated with HCV, highlighting the importance of continued sexual health services and prevention efforts during such outbreaks.

## Introduction

Compared to the general population, increased risk of hepatitis C virus (HCV) acquisition has been observed among men who have sex with men (MSM) with HIV and, due to similarities in transmission networks and behaviours, MSM using HIV preexposure prophylaxis (PrEP) [1–3]. Among MSM in particular, HCV transmission occurs predominantly through sexual contact (e.g., receptive condomless anal sex (CAS) or unprotected fisting) and in settings where sexual disinhibition is present (i.e., engaging in group sex or chemsex) [4,5].

The COVID-19 and mpox outbreaks led to substantial lifestyle restrictions. For COVID-19, these restrictions included restricted access to public spaces, wearing face masks, cancelling large events, restricting international travel, curfews and minimizing physical contact, also with sexual partners [6]. In response to the mpox outbreak, public health control measures included offering vaccination, increasing awareness through health communication, sharing information and promoting behaviours that reduce the risk of mpox transmission [7,8]. In addition, it was recommended to refrain from physical contact when mpox was diagnosed. The measures implemented during the COVID-19 and mpox epidemics, as well as their associated public health awareness campaigns, may have led to changes in sexual and drug use behaviours [9–11]. For COVID-19, these measures have influenced the transmission of sexually transmitted infections (STI), leading to a significant lower STI incidence rate compared to pre-COVID periods [12]. However, data on behaviours specifically related to HCV acquisition during these epidemics are scarce and could be important in gauging the HCV epidemic among MSM at increased risk of HCV.

The Interventions to Curb Hepatitis C Virus Reinfection Among MSM (ICECREAM) clinical trial was aimed at establishing interventions that could effectively decrease behavioural risk associated with HCV among MSM who had cleared HCV infection [13]. This study contained a prerandomisation phase of 6 months during which participants received standard care with no intervention. Data collection occurred during and after the COVID-19 and mpox epidemics. In this study, our aim was to assess the impact of the COVID-19 and mpox outbreaks, separately, on sexual and drug use behaviours associated with HCV infection.

## Methods

### Study design and population

We conducted a prospective, longitudinal analysis using data from MSM enrolled in the ICECREAM study. The ICECREAM study is an international, multicentred, phase 2, 3-arm, randomized trial enrolling MSM with and without HIV who had a confirmed previous HCV infection, were successfully treated or spontaneously cleared the virus, and are at risk for HCV reinfection. After inclusion, individuals were followed in routine care for six months during a control, prerandomisation phase, after which they were randomized 1:1:1 to one of the following three arms: an online risk-reduction behavioural intervention, a home-based HCV-RNA self-sampling test service, or a combination of both. Participants were then followed for 18 months during the intervention. The trial enrolled participants between 7 September 2021 and 29 February 2024 (7 September 2021–24 November 2023 in the Netherlands, 30 January 2023–29 February 2024 in France). Participant follow-up is currently ongoing. Detailed study procedures have been described elsewhere [13].

For this study, we included all individuals who completed at least one questionnaire during the prerandomisation phase and answered questions on the impact of COVID-19 restrictions or mpox outbreak on sexual and drug use behaviours.

### Study procedures and outcomes

At enrolment and month six, participants were asked to complete an online questionnaire with questions about sociodemographic characteristics, clinical outcomes and detailed sexual and drug use behaviour, all of which were self-reported. Sociodemographic characteristics included age at study visit, region of origin and educational level (≤high school degree vs. >high school degree). Clinical outcome data included information about HIV status, current HIV treatment (for participants with HIV), PrEP use (for participants without HIV), previous HCV infections, HCV treatment and STI diagnosis in the last six months. Information about sexual and drug use behaviour referred to the preceding six months and included the following: stable partner (yes/no), number of casual partner(s), CAS acts (yes/no), unprotected fisting (yes/no), sharing of sex toys (yes/no), sharing of straws when snorting drugs (yes/no), injection drug use (IDU) (yes/no), group sex (yes/no), sexualised drug use (SDU) (yes/no), and chemsex (yes/no). We defined SDU as the use of any drug before or during sex, excluding alcohol, and chemsex as the use of methamphetamine, γ-hydroxybutyric acid (GHB)/γ-butyrolactone (GBL) or mephedrone before or during sex. We also calculated the HCV-MOSAIC risk score [5,14]. This score was previously developed and validated for primary early HCV infection and HCV reinfection and is calculated by adding up the *β* coefficients of the following six behaviours, when present: receptive CAS (*β* = 1.1), sharing of sex toys (*β* =1.2), unprotected fisting (*β* = 0.9), sharing of straws (*β* = 1.0), IDU (*β* = 1.4) and having an ulcerative STI (*β* = 1.4). The score gives a range between 0 and 7 and individuals with a risk score ≥2.0 were considered behaviourally susceptible for HCV reinfection [5,14].

Questions about the influence of COVID-19 restrictions on sexual and drug use behaviour were included in the questionnaire at the beginning of the study (i.e., September 2021) and of the mpox outbreak from November 2022 onwards, all referring back to the preceding six months (Supplementary Figure 1, Supplemental Digital Content for the COVID-19 stringency and implemented mpox measures over time [15]). The six-month reference periods for assessing the impact of the COVID-19 restrictions on behaviour overlapped with the roll-out of COVID-19 vaccination campaigns, which began in early 2021 in both the Netherlands and France. For the impact of the mpox outbreak, the reference periods overlapped with the early phase of the mpox outbreak, starting in May 2022 in both countries. In the questionnaire, both topics were introduced and questions were worded as follows: *“Did the COVID-19 effect measures have an effect on your sex life or substance use in the past six months?”* and *“Did mpox have an effect on your sex life or substance use in the past six months?”* For both questions, respondents could select one of the three response options: “*No*”, “*Yes*” or, in case of the impact of COVID-19 effect measures, “*Not applicable, there were no COVID-19 restrictions*”. If participants responded “*Yes*”, additional questions were asked about whether the outbreak had an impact on number of sex partners, number of receptive CAS acts, number of group sex activities, number of chemsex activities, number of times sex toys were used, number of times fisting occurred (fisted or were fisted), and number of times drugs were injected.

### Statistical analyses

Characteristics of participants at enrolment were described both overall and within country, and compared between countries using Pearson's *χ*^2^ or Fisher's exact test for categorical variables and Student's *t*-test or Mann–Whitney *U* test for continuous variables.

We examined changes over calendar time for the following outcomes: proportion of persons whose behaviours were influenced by COVID-19 restrictions, proportion of persons whose behaviours were influenced by the mpox outbreak, mean HCV-MOSAIC risk score, and proportion of persons engaging in each of the individual risk behaviours included in the risk score. We modelled binomial outcomes using logistic regression and continuous outcomes using linear regression. We accounted for repeated measures using either a random-intercept for participant or clustered variance estimators. In all models, we added calendar time as a continuous covariate, which was modelled using restricted cubic splines with three knots (at the 25^th^, 50^th^, and 75^th^ percentiles). From these models, we plotted the predicted probability (for binomial outcomes) or average (for continuous outcomes) over calendar time along with its 95% confidence intervals (CI). We assessed for changes in behaviours across calendar time by testing the spline-derived covariates using Wald X^2^ tests. To assess whether changes in outcomes were different between countries as severity of mitigation measures differed between the two countries (Supplementary Figure 1, Supplemental Digital Content), we additionally ran models including an interaction term between country of enrolment in the study and calendar time, which was tested using a Wald X^2^ test.

We then assessed the determinants of changes in behaviour from COVID-19 restrictions or the mpox outbreak by modelling these outcomes using logistic regression, while including individual determinants as covariates and accounting for repeated measures, as described above. The determinants evaluated were as follows: age (continuous), HIV status (positive vs. negative), number of previous HCV infections (1 vs. ≥2), educational level (≤high school degree vs. >high school degree), combined effect of group sex and chemsex (no/yes), and the HCV-MOSAIC risk score (continuous, range 0–7). Univariable odds ratios (OR) comparing the odds of change in behaviours between levels of determinants and their 95%CI were calculated. We then included all determinants in univariable analysis *a priori* to construct a multivariable model. Participants with missing data on any variable in the model were excluded.

Statistical analyses were conducted using Stata (v17.0, StataCorp, College Station, TX, USA).

## Results

### Description of the study population

Between 7 September 2021 and 29 February 2024, 258 MSM were enrolled in the ICECREAM study. Of them, 24 (9.3%) were excluded from the analysis because they either withdrew from the study before completing any of the questionnaires or did not complete at least 1 questionnaire before randomization (Supplementary Figure 2, Supplemental Digital Content). Among those who completed at least 1 questionnaire before randomization, 220 participants answered questions about the impact of COVID-19 (*n* = 220) and/or mpox measures (*n* = 146) on behaviours and thus were included in the current analysis (the number of questionnaires completed by calendar month overall and stratified by country of enrolment is presented in Supplementary Table 1, Supplemental Digital Content).

Of those who were included in the analysis, 208 (95.0%) MSM completed the questionnaire at enrolment and are described in Table 1, both overall and stratified by country of enrolment. The median age of participants at enrolment was 51 years [interquartile range (IQR) = 45–58] and the majority had obtained a degree higher than high school (*n* = 161, 77.4%). 171 (82.2%) participants received an HIV diagnosis prior to enrolment and 33 (15.9%) reported the use of HIV PrEP. The median HCV-MOSAIC risk score at baseline was 2.3 (IQR = 1.1–3.5), with 133 (63.9%) MSM having an HCV-MOSAIC risk score ≥2.0 (i.e., high susceptibility for HCV acquisition). Compared to participants in France, those in the Netherlands reported group sex less often (*P* < 0.001). There were no other differences in demographic, clinical and behavioural characteristics of participants between countries.

**Table 1:** Characteristics of included MSM participating in the ICECREAM study at baseline, overall and stratified by country of enrolment, 2021–2024.

			Country of enrolment				
			
	Overall^a^ (*n* = 208)		The Netherlands (*n* = 111)		France (*n* = 97)		
				
	*n* ^b^	%^b^	*n* ^b^	%^b^	*n* ^b^	%^b^	*P*
*Sociodemographic characteristics*							
Age, years							
Median [IQR]	51	[45–58]	52	[45–59]	51	[44–57]	0.43
≤34	12	5.8%	7	6.3%	5	5.2%	0.48
35-44	40	19.2%	18	16.2%	22	22.7%	
≥45	156	75.0%	86	77.5%	70	72.2%	
Higher than high school degree	161	77.4%	86	77.5%	75	77.3%	0.98
*HIV and HCV clinical characteristics*							
HIV status or PrEP use							0.36
Positive	171	82.2%	90	81.1%	81	83.5%	
Negative and using PrEP	33	15.9%	20	18.0%	13	13.4%	
Negative and not using PrEP	4	1.9%	1	0.9%	3	3.1%	
Years since HIV diagnosis^c^							
Median [IQR]	17.8	[12.8–23.5]	16.3	[12.8–21.2]	19.7	[12.8–26.7]	0.11
On ART^c^	171	100.0%	90	100.0%	81	100.0%	1.00
Number of previous HCV infections							0.48
1	159	76.4%	87	78.4%	72	74.2%	
≥2	49	23.6%	24	21.6%	25	25.8%	
Years since last HCV diagnosis							
Median [IQR]	5.0	[2.2–9.8]	5.9	[3.0–10.3]	4.7	[1.7–9.4]	0.12
Treatment of last HCV infection							0.41
Pegylated-interferon based	81	38.9%	44	39.6%	37	38.1%	
DAA based	104	50.0%	52	46.9%	52	53.6%	
No treatment (spontaneous clearance)	23	11.1%	15	13.5%	8	8.3%	
*Sexual and drug use behaviours* ^6M^							
Stable sexual partner	93	44.7%	49	44.1%	44	45.4%	0.86
Casual partner	172	82.7%	90	81.1%	82	84.5%	0.51
Number of casual partner(s)							
Median [IQR]	10	[3–30]	10	[2–25]	12	[4–35]	0.13
Receptive CAS	173	83.2%	91	82.0%	82	84.5%	0.62
Unprotected fisting	82	39.4%	42	37.8%	40	41.2%	0.62
Sharing of sex toys	58	27.9%	35	31.5%	23	23.7%	0.21
Sharing of straws	70	33.7%	41	36.9%	29	29.9%	0.28
IDU	37	17.8%	19	17.1%	18	18.6%	0.79
Any STI	79	38.0%	38	34.2%	41	42.3%	0.23
Ulcerative STI^d^	49	23.6%	23	20.7%	26	26.8%	0.30
SDU^e^	139	66.8%	76	68.5%	63	65.0%	0.59
Chemsex^f^	114	54.8%	59	53.2%	55	56.7%	0.61
Group sex	118	56.7%	47	42.3%	71	73.2%	<0.001
HCV-MOSAIC risk score							
Median [IQR]	2.3	[1.1–3.5]	2.3	[1.1–3.5]	2.1	[1.1–3.4]	0.75

*P*-value represents the statistical comparison between country of enrolment using Pearson's χ^2^ or Fisher's exact test for categorical variables and Student's *t*-test or Mann–Whitney *U* test for continuous variables. 6M, in the previous 6 months; ART, Antiretroviral therapy; CAS, Condomless anal sex; DAA, Direct acting antiviral; HCV, Hepatitis C virus; HIV, Human immunodeficiency virus; ICECREAM, Interventions to curb hepatitis C reinfection among MSM; IDU, Injecting drug use; IQR, Interquartile range; MOSAIC, MSM Observational Study of Acute Infection with HCV; MSM, Men who have sex with men; PrEP, preexposure prophylaxis; SDU, Sexualised drug use; STI, Sexually transmitted infection.

a In total, 220 participants were included in the analysis of which 208 completed the baseline questionnaire.

b Unless otherwise indicated.

c Only among those with HIV.

d Having a syphilis, herpes or Lymphogranuloma Venereum infection at study visit.

e Defined as the use of any drugs before or during sex, excluding alcohol.

f Defined as the use of methamphetamine, γ-hydroxybutyric acid (GHB)/γ-butyrolactone (GBL) and/or mephedrone before or during sex.

### Effect of COVID-19 restrictions on sexual behaviour

**Fig. 1:**
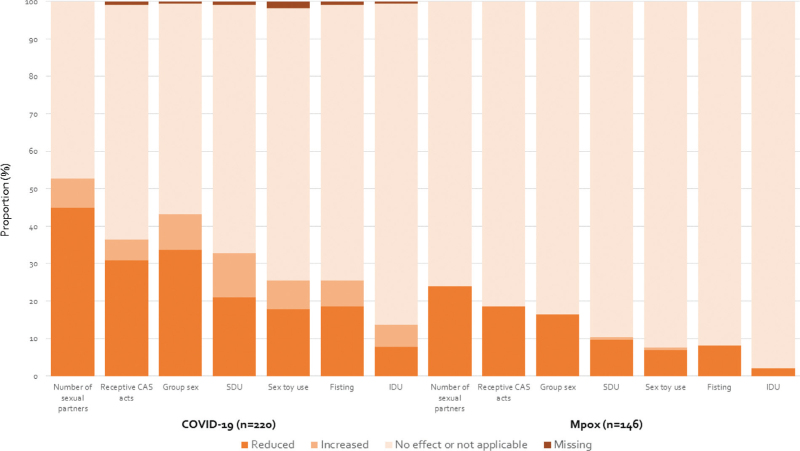
Overall cumulative proportion of MSM who reported a reduction in sexual and drug use behaviours impacted by the COVID-19 restrictions and mpox measures in the past six months in the period September 2021–September 2024.

**Fig. 2:**
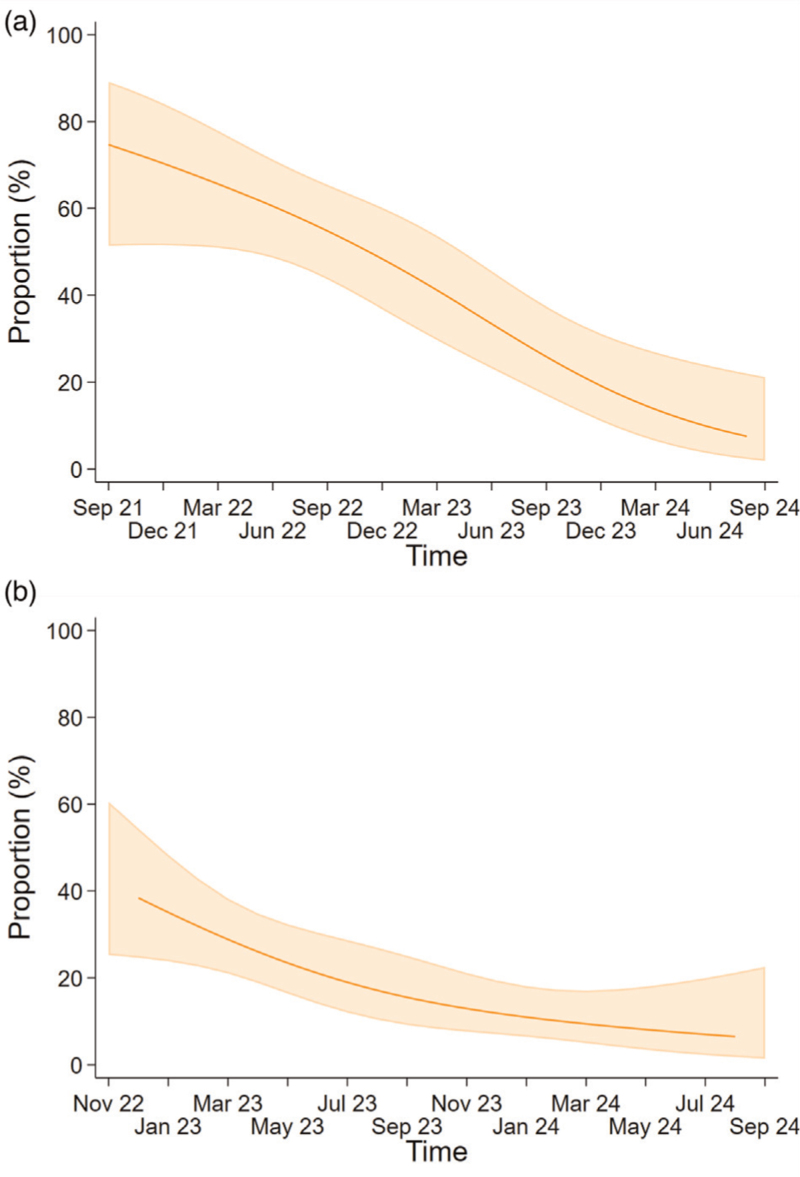
Proportion reporting any effect of (a) COVID-19 restrictions and (b) mpox on sexual and drug use behaviour associated with HCV in the preceding six months over calendar time among included MSM.

Overall, 125 of 220 (56.8%) participants ever reported that their sexual or drug use behaviours in the previous six months were affected by COVID-19 restrictions, with the majority reporting that the restrictions had reduced their number of sexual partners (99/220, 45.0%) (Fig. 1). The predicted proportion whose behaviours were affected by COVID-19 restrictions was 74.7% in September 2021 and gradually decreased to 6.7% in September 2024 (Fig. 2A). Over calendar time, the probability of reporting any impact of COVID-19 restrictions on sexual behaviours significantly decreased (*P* < 0.001), and did not differ between countries of enrolment (*P* for interaction = 0.24) (Supplementary Figure 3, Supplemental Digital Content). In multivariable analysis, older age was significantly associated with an increased probability of change in behaviour (adjusted OR per 10 years = 1.59, *P* = 0.03) (Supplementary Table 2, Supplemental Digital Content).

CAS, Condomless anal sex. IDU, Injecting drug use; SDU, Sexualised drug use.

Fitted solid line represents the predicted proportions per calendar month. The shaded area surrounding each line represents the 95% confidence interval. HCV, Hepatitis C virus; MSM, Men who have sex with men.

### Effect of the mpox outbreak on sexual behaviour

Of the 220 MSM included in the COVID-19 analysis, 146 MSM completed the questionnaire after November 2022 and contributed to the analysis about the impact of the mpox outbreak on sexual and drug use behaviour. Compared to those excluded from the mpox analysis, included MSM were more often enrolled in French participating centres (*P* < 0.001) and more often reported engaging in group sex activities (*P* = 0.001) (Supplementary Table 3, Supplemental Digital Content). Between November 2022 and September 2024, 39 of 146 (26.7%) participants ever reported that their sexual and drug use behaviours in the previous six months were affected by the mpox outbreak. The majority reported that the outbreak led to a reduced number of sexual partners (35/146, 23.9%) and number of receptive CAS acts (27/146, 18.5%) (Fig. 1). The predicted proportion who reported that their behaviours were affected by the mpox outbreak was 41.9% in November 2022 and declined to 6.0% in September 2024 (Fig. 2n). Over calendar time, the probability of reporting any impact of the mpox outbreak on sexual and drug use behaviours significantly decreased (*P* = 0.001). The test for interaction between calendar time and country was precluded by the few participants enrolled in the Netherlands after the start of the mpox outbreak (Supplementary Table 1, Supplemental Digital Content). Multivariable analyses showed no associations between any of the determinants and reporting an impact of mpox on behaviours (Supplementary Table 4, Supplemental Digital Content).

### Sexual and drug use behaviours over calendar time

**Fig. 3:**
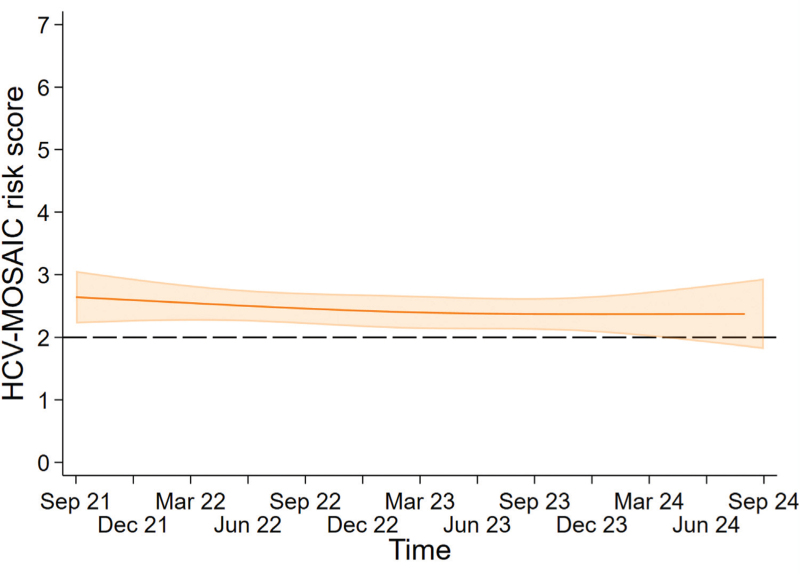
Average predicted HCV-MOSAIC risk score over time among included MSM between September 2021 and September 2024.

Between September 2021 and September 2024, the average predicted HCV-MOSAIC risk score was consistently above 2.0. Over calendar time, the average HCV-MOSAIC risk score remained constant over time (*P* = 0.59) (Figure 3) and did not differ between countries of enrolment (*P* for interaction = 0.15) (Supplementary Figure 4, Supplemental Digital Content). Supplementary Figure 5, Supplemental Digital Content provides the predicted proportions of MSM reporting individual risk behaviours over calendar time. Overall, the predicted probability of reporting receptive CAS was the highest followed by unprotected fisting. Between September 2021 and September 2024, we observed no significant changes in the predicted proportions for any of the individual risk behaviours (receptive CAS: 82.4–79.2%, *P* = 0.92; unprotected fisting: 42.5–47.0%, *P* = 0.57; sharing of toys: 33.7–34.5%, *P* = 0.47; sharing of straws: 39.5–39.7% *P* = 0.54; IDU: 10.6–9.7%, *P* = 0.23; having an ulcerative STI: 13.2–22.4% *P* = 0.38).

Fitted solid line represents the average predicted HCV-MOSAIC risk per calendar month. The shaded area surrounding the line represents the 95% confidence interval. The dashed black line represents the HCV-MOSAIC risk score cut-off, with a risk score ≥2.0 indicating high risk of HCV infection. HCV, Hepatitis C virus; MOSAIC, MSM Observational Study of Acute Infection with HCV; MSM, Men who have sex with men.

## Discussion

In this study, we evaluated the impact of both the COVID-19 and mpox outbreak on sexual and drug use activity, as it specifically relates to HCV infection, in the Netherlands and France. We observed that participants more frequently reported that their sexual and drug use behaviours were affected early on after COVID-19 restrictions were implemented (75%) and after the first cases of mpox (42%) were identified when compared to later periods. Nevertheless, over time, the average HCV-MOSAIC risk score remained stable and was consistently ≥2.0, indicating high HCV reinfection risk.

The recruitment period and follow-up during the prerandomisation phase overlapped with periods of COVID-19 restrictions. Although some of these restrictions might have differed between the Netherlands and France, restrictions that could imply differences in HCV-related behaviours were largely similar. Participants did report an effect of COVID-19 restrictions on their sexual and drug use behaviours: of the possible precautions, they most commonly reported a reduction in the number of sexual partners. Decreasing numbers of sexual partners during the COVID-19 epidemic has been observed in previous studies among PrEP-using MSM in the Netherlands, France and elsewhere [10,16–18]. In general, individuals of advanced age or with HIV were considered as being at high risk of severe illness from COVID-19 [19,20]. Indeed, our study showed that older participants more often reported that COVID-19 restrictions affected their behaviours compared to younger individuals. However, we did not observe an association with HIV status. In January and February 2022, some major restrictions were removed, likely corresponding to the decrease in the proportion of individuals reporting effects of COVID-19 on behaviours observed in our study.

In both countries, the number of reported mpox cases peaked in July 2022 followed by a rapid decline in the months thereafter [7,21,22]. Results from an online survey in a convenience sample of MSM in the Netherlands and among MSM with HIV or using PrEP in France showed that the majority considered themselves to be at risk, were concerned about acquiring mpox or thought mpox symptoms were severe [23,24]. It is possible that these perceptions led to changes in sexual behaviours. Again, most individuals who did change their behaviours reported fewer numbers of sexual partners, which is in line with previous studies conducted among MSM in Europe and the Americas [11]. Shortly after the first cases of mpox were confirmed, more information became available on the risks of mpox exposure and symptoms. Vaccination campaigns were then instated in both countries, which additionally contributed to changes in the mpox epidemic [7,25]. Since then, new cases of mpox were infrequent, but some sporadic outbreaks in other countries, including France, have been reported [26]. The rapid decline in new confirmed cases of mpox and vaccination-induced immunity likely resulted in the decline in the proportion of individuals reporting any effect of mpox on sexual and drug use behaviours.

Despite the reported reductions in the numbers of sexual partners during both outbreaks, participants maintained high and stable levels of behavioural risk for HCV reinfection, as determined by the HCV-MOSAIC score [5,14], and reported a median of ten casual partners at baseline, which is associated with increased susceptibility of HCV acquisition [4]. When examining individual behaviours in the HCV-MOSAIC score, receptive CAS and unprotected fisting were the more common behaviours driving the HCV-MOSAIC score above the threshold for high risk of HCV acquisition (i.e., ≥2.0). Our findings may suggest that even if MSM might have reduced their number of partners, with those that they did have sex with, they likely continued to engage in sexual and drug use activities. During the COVID-19 outbreak, this may have occurred in private or informal settings due to the closure of public venues (e.g., gay saunas, nightclubs, etc.) [27]. However, it remains debateable whether these behaviours during the COVID-19 or mpox epidemics translated into higher risk of HCV. Newly diagnosed HCV reinfections were rarely observed during the COVID-19 epidemic in MSM in the Netherlands and in Berlin, Germany [28,29]. Nevertheless, these observations are in light of the massive reductions in HCV testing among MSM with HIV during the COVID-19 epidemic and a lower number of PrEP-care consultations and STI testing, among MSM using PrEP compared to pre-COVID periods [12,30].

To our knowledge, this is one of the very few studies among MSM at risk of an HCV reinfection with detailed data on sexual and drug use behaviours related to HCV acquisition during these two outbreaks. However, this study is not without limitations. First, questions about engaging in sexual and drug use behaviours in the previous six months were self-reported and could therefore be influenced by individual recall. Second, outcomes of this study may not be generalizable to the wider MSM community. Participants in this study were in care at an HIV treatment centre or seeking care at a Centre for Sexual Health, and previously had an HCV infection. Therefore, this study population reflects mostly those at risk of HCV reinfection who commonly visit these places for sexual healthcare. Third, no data was collected on the uptake of COVID-19 or mpox vaccination. MSM who had received complete or partial vaccination might have felt more comfortable to engaging in sexual activities compared to those who were unvaccinated. Fourth, we did not assess the incidence of HCV and thus could not evaluate whether changes in behaviours also led to changes in HCV reinfections. Fifth, the HCV-MOSAIC risk score was developed using data from a period when HCV treatment was primarily interferon-based. As HCV incidence has decreased with the widespread availability of direct-acting antivirals, re-evaluation of the risk score in the current context is recommended. Lastly, there was limited overlap in recruitment periods between countries and the small number of completed questionnaires on the effect of mpox from the Netherlands due to the earlier start of the study in the Netherlands and thus the end of the prerandomisation follow-up period, which made any assessment of between-country differences not possible.

In conclusion, changes in sexual and drug use behaviours affected by the COVID-19 and mpox outbreak were predominantly apparent when COVID-19 restrictions and mpox measures were in place but waned over time when COVID-19 restrictions were released, COVID-19 and mpox preventive vaccination was offered and more information on mpox exposure and vaccine effectiveness became available. Future research should evaluate if these changes in sexual and drug use behaviours associated with HCV had any impact on testing rates and subsequent HCV reinfections. The consistently high behavioural risk reported over time during the COVID-19 and mpox outbreaks, highlight the importance of continued sexual health services and prevention measures during such outbreaks.

## Acknowledgements

The authors thank all participants of the ICECREAM study. In addition, the authors thank the members of the ICECREAM advisory board and community members who contributed to the ICECREAM study and IMEA (Institut de Médecine et d’Epidémiologie Appliquée) for the administrative management of the study in France.

The collaborators of the ICECREAM study are: M. van der Valk, J. Schinkel, S. Rebers, F. Pijnappel, H. van Eden (Amsterdam UMC, Academic Medical Centre of the University of Amsterdam, Amsterdam, the Netherlands). J. Stalenhoef, F. van Malsem, R. van Heerde (OLVG, Amsterdam, the Netherlands). M. van der Valk, H. Nobel, W. Alers, L. Elsenburg (DC Clinics Lairesse, Amsterdam, the Netherlands). D. Verhagen, F. Lauw, M. van Wijk (Medical Centre Jan van Goyen, Amsterdam, the Netherlands). J. den Hollander, A Brouwer (Maasstad Ziekenhuis, Rotterdam, the Netherlands). E. Leyten, S. Wildenbeest (Haaglanden Medical Centre, The Hague, the Netherlands). T. Mudrikova (University Medical Centre Utrecht, Utrecht, the Netherlands). K. Hage, A. Boyd, U. Davidovich, A. Matser, E. Generaal, E. Hoornenborg, M. Prins, M. van der Kerkhof, C. Kips, L. Flobbe, F. Mouthaan, S. Elzinga, D. Loomans, E. Ersan, K. Yap, K. de Jong, I. Peters, S. de Graaf (Public Health Service of Amsterdam, Amsterdam, the Netherlands). Paul Zantkuijl, Ejay de Wit (Soa Aids Nederland). H. Rougier, T. Melnyk, K. Lacombe, T. Chiarabini, B. Lefevbre, N. Valin, Z. Ouazene, Y. Abi Aad, E. Rougier, A. Goetschel, D. Bollens, R. Chruchet (Saint Antoine hospital, Paris, France). M. Valantin, L. Schneider, R. Palich, R. Tubiana, A. Faycal, E. Bourzam, M. Favier, O. Derradji (Pitié-Salpêtrière Hospital, Paris, France). G. Pialoux, J. Chas, C. Palacios, M. Siguier, N. Sandid (Tenon hospital, Paris, France). P. Campa, J. Lourenco, J. Krause, S. Seang (Maison Médicale Chemin Vert, Paris, France).

Authors’ contributions: K.H. contributed to the conception and design of the work, the data management, data analysis and drafted the manuscript. A.B. contributed to the conception and design of the work and interpretation of data. U.D., E.G., E.H., M.vdV., J. Schinkel and P.Z. contributed to the conception and design of the work. M.vdV., D.V., J. Stalenhoef, J.dH., E.L., T.M., H.R., M.V., G.P., and P.C. contributed to acquisition of data. K.L. is principal investigator of the ICECREAM study in France. M.P. is the principal investigator of the ICECREAM study in the Netherlands and contributed substantially to the conception and design of the work and interpretation of data. All authors critically revised the manuscript, and all approved the final version.

Data availability statement: The ICECREAM data are owned by the Public Health Service of Amsterdam, the Netherlands. Original data can be requested by submitting a research proposal to the Principal Investigator M. Prins (mprins@ggd.amsterdam.nl).

Funding statement: This work was supported by The Netherlands Organisation for Health Research and Development (ZonMw; grant number 522004006); the ANRS | Maladies émergentes (grant number ECTZ108101); and GGD research funds.

Ethical approval statement: The ICECREAM study has been approved by the Medical Research Ethics Committee of the Amsterdam University Medical Centres in the Netherlands (registration number: NL68718.018.19), *Comité de Protection des Personnes* of Hôpital Tarnier-Cochin in France (registration number: 2022-A00533-40) and ethical committees/board of directors of each recruiting institute. All participants gave written informed consent for participation.

Clinical trial registration: This trial is registered with ClinicalTrials.gov (number NCT04156945).

### Conflicts of interest

A.B. has received a speakers fee from Gilead Sciences, independent for the submitted work. EH's institution has received independent scientific support from Gilead Sciences. M.vdV.'s institution has received consultancy fees from Gilead, MSD and ViiV outside the submitted work and research grants from Gilead, Merck Sharp Dome and ViiV. J.dH. has received consultancy fees from Gilead, MSD and ViiV outside the submitted work. T.M.'s institution has received consultancy fees from ViiV, Gilead and MSD, and research grants from ViiV and Gilead, unrelated to the submitted work. K.L. has received speakers fees and independent scientific support from Gilead Sciences, MSD, and ViiV Healthcare, outside the submitted work. MP's institution has received speakers fees and independent scientific support from Gilead Sciences, Roche, MSD, and Abbvie, outside the submitted work. All other authors report no potential conflicts.

## Supplementary Material

Supplemental Digital Content
