# Supplementary material for: The impact of the COVID-19 and mpox outbreaks on behaviours associated with HCV infection among MSM: results from the prerandomisation phase of a clinical trial
Source: AIDS. 2025 Jun 20;39(12):1803–12. doi: 10.1097/QAD.0000000000004265 (PMC12404629; doi:10.1097/QAD.0000000000004265)
Supplement: Supplemental Digital Content [file aids-39-1803-s001.docx]

**SUPPLEMENTARY MATERIALS**

**The impact of the COVID-19 and mpox outbreaks on behaviours associated with hepatitis C virus infection among men who have sex with men: results from the pre-randomization phase of a clinical trial**

Kris Hage, Anders Boyd, Udi Davidovich, Ellen Generaal, Elske Hoornenborg, Paul Zantkuijl, Marc van der Valk, Dominique Verhagen, Janneke Stalenhoef, Jan den Hollander, Eliane Leyten, Tania Mudrikova, Hayette Rougier, Thibault Chiarabini, Marc-Antoine Valantin, Gilles Pialoux, Pauline Campa, Janke Schinkel, Karine Lacombe, Maria Prins on behalf of the ICECREAM study group

**Content**

[**Supplementary Figure 1.** COVID-19 stringency index and implemented mpox measures between March 2021 and December 2022 in the Netherlands and France [1]. 2](#_Toc198622798)

[**Supplementary Figure 2.** Flowchart of study participants. 4](#_Toc198622799)

[**Supplementary Table 1.** Number of completed questionnaires per calendar month among included MSM between September 2021 and September 2024 5](#_Toc198622800)

[**Supplementary Figure 3.** Proportion reporting any effect of COVID-19 restrictions on sexual and drug use behaviour associated with HCV in the preceding six months over calendar time stratified by country of enrolment among included MSM between September 2021 and September 2024. 6](#_Toc198622801)

[**Supplementary Table 2.** Determinants of reporting any effect of the COVID-19 restrictions among MSM enrolled in the ICECREAM study between September 2021 and September 2024. 7](#_Toc198622802)

[**Supplementary Table 3.** Comparison of characteristics at baseline of MSM who are included vs. excluded in the analyses of the impact of mpox measures on sexual and drug use behaviours between 2021-2024. 8](#_Toc198622803)

[**Supplementary Table 4.** Determinants of reporting any effect of the mpox outbreak among MSM enrolled in the ICECREAM study between November 2022 and September 2024. 10](#_Toc198622804)

[**Supplementary Figure 4**. Average predicted HCV-MOSAIC risk score over time stratified by country of enrolment among included MSM between September 2021 and September 2024. 11](#_Toc198622805)

[**Supplementary Figure 5.** Predicted proportion reporting individual risk behaviours included in the HCV-MOSAIC risk score in the preceding six months over calendar time among included MSM between September 2021 and September 2024 [9, 10]. 12](#_Toc198622806)

[**References** 13](#_Toc198622807)

**
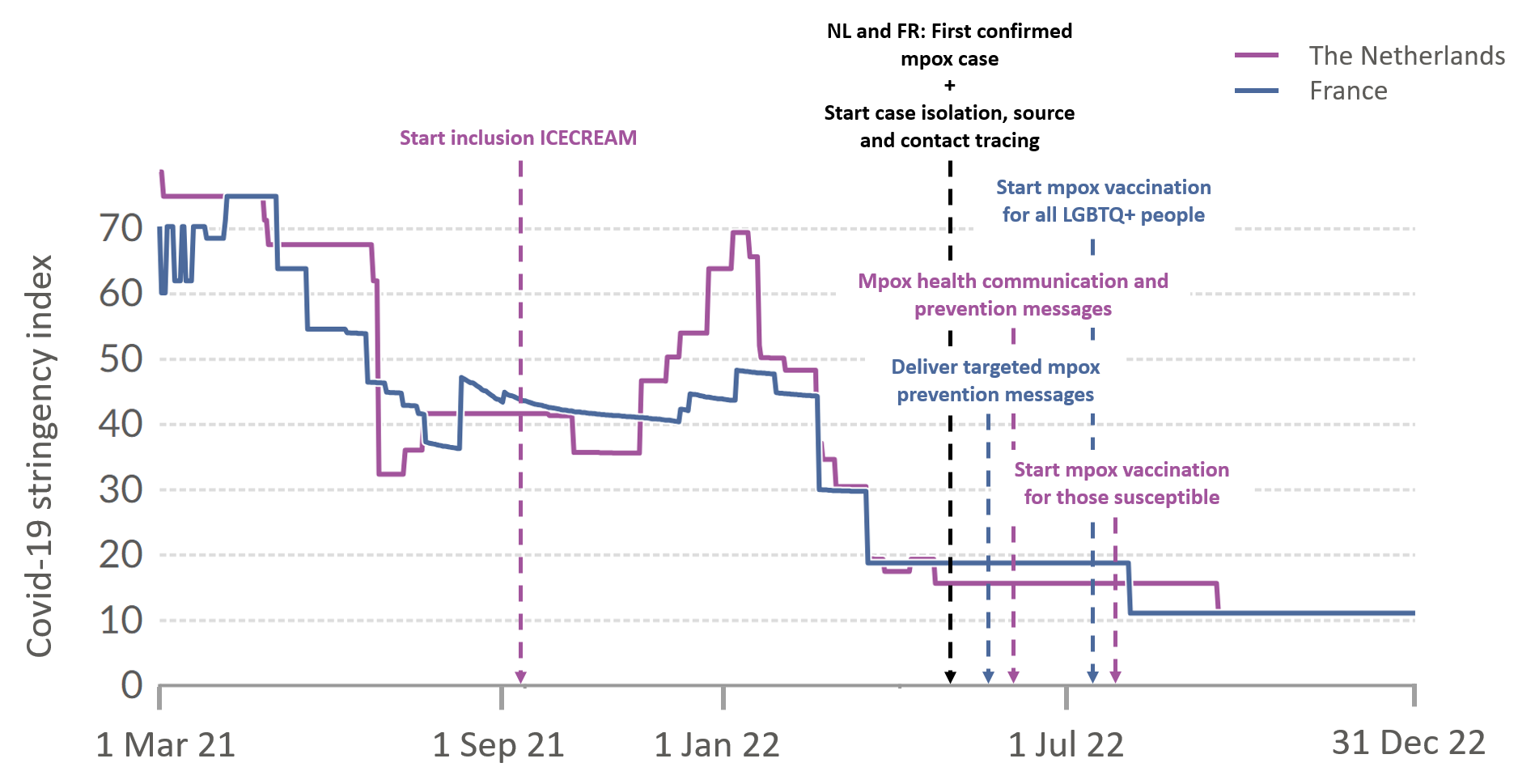
**

**Supplementary Figure 1.** COVID-19 stringency index and implemented mpox measures between March 2021 and December 2022 in the Netherlands and France [1]. *Participant recruitment started in September 2021 in the Netherlands. Shortly thereafter, the SARS-CoV-2 Omicron variant was rapidly spreading across parts of Europe, including the Netherlands and France [2, 3]. In December 2021, the Dutch government imposed a second lockdown where only essential facilities, such as food stores and pharmacies, remained open [4]. In France, the lockdown was less stringent and included the closure of night clubs and cancelling of indoor gatherings of >2000 people and outdoor gathering >5000 people [5]. In January and February 2022, both countries lifted the restrictions to finally transition to an endemic phase [4]. In both countries, the first case of mpox was confirmed on 20 May 2022 [6]. In response to the mpox outbreak, both countries immediately implemented case isolation and contact tracing [7, 8]. In addition, the French authorities performed preventive actions to increase awareness and offered mpox vaccination to all Lesbian, Gay, Bisexual, Transgender, Queer/Questioning plus individuals with identities not captured within the acronym who reported multiple sexual partners or sex work and could also be considered on the individual level depending on exposure [8]. In the Netherlands, health communication and prevention messages were disseminated via social media and community platforms. Vaccination for mpox was only offered to men who have sex with men who were susceptible for mpox acquisition, including those using pre-exposure prophylaxis or those who were involved in frequent sexual activity or group sex [7]. Shortly thereafter, confirmations of new mpox cases declined to a minimum in September 2022 in the Netherlands followed by France in October the same year [6].*

**
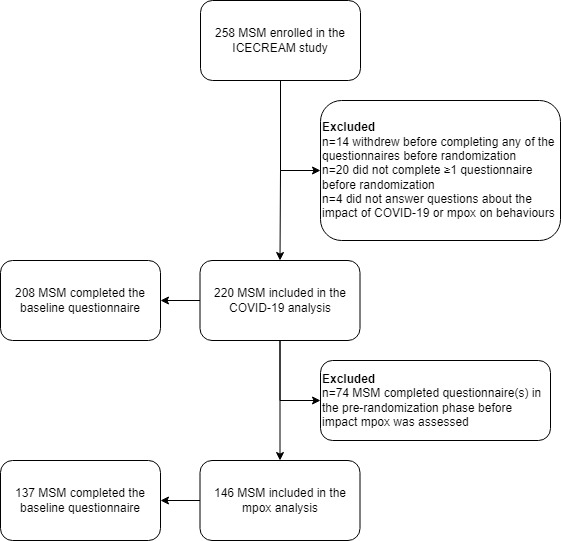
**

# **Supplementary Figure 2.** Flowchart of study participants.

Abbreviations: ICECREAM, Interventions to curb hepatitis C reinfection among MSM; MSM, Men who have sex with men

# **Supplementary Table 1.** Number of completed questionnaires per calendar month among included MSM between September 2021 and September 2024

|  | **Number of completed questionnaires on risk behaviours and impact of COVID-19 restrictions** | | | **Number of completed questionnaires on impact of mpox outbreak**^†^ | | |
| --- | --- | --- | --- | --- | --- | --- |
| **Calendar month** | **Total (n=220)** | **The Netherlands**  **(n=117)** | **France (n=103)** | **Total (n=146)** | **The Netherlands (n=43)** | **France (n=103)** |
| Sep 2021 | 19 | 19 | 0 |  |  |  |
| Oct 2021 | 7 | 7 | 0 |  |  |  |
| Nov 2021 | 6 | 6 | 0 |  |  |  |
| Dec 2021 | 1 | 1 | 0 |  |  |  |
| Jan 2022 | 2 | 2 | 0 |  |  |  |
| Feb 2022 | 5 | 5 | 0 |  |  |  |
| Mar 2022 | 23 | 23 | 0 |  |  |  |
| Apr 2022 | 21 | 21 | 0 |  |  |  |
| May 2022 | 16 | 16 | 0 |  |  |  |
| Jun 2022 | 12 | 12 | 0 |  |  |  |
| Jul 2022 | 4 | 4 | 0 |  |  |  |
| Aug 2022 | 11 | 11 | 0 |  |  |  |
| Sep 2022 | 4 | 4 | 0 |  |  |  |
| Oct 2022 | 21 | 21 | 0 |  |  |  |
| Nov 2022 | 12 | 12 | 0 | 7 | 7 | 0 |
| Dec 2022 | 10 | 10 | 0 | 10 | 10 | 0 |
| Jan 2023 | 4 | 4 | 0 | 4 | 4 | 0 |
| Feb 2023 | 10 | 4 | 6 | 10 | 4 | 6 |
| Mar 2023 | 25 | 3 | 22 | 25 | 3 | 22 |
| Apr 2023 | 18 | 6 | 12 | 18 | 6 | 12 |
| May 2023 | 8 | 2 | 6 | 8 | 2 | 6 |
| Jun 2023 | 11 | 2 | 9 | 11 | 2 | 9 |
| Jul 2023 | 8 | 4 | 4 | 8 | 4 | 4 |
| Aug 2023 | 12 | 1 | 11 | 12 | 1 | 11 |
| Sep 2023 | 14 | 1 | 13 | 14 | 1 | 13 |
| Oct 2023 | 23 | 4 | 19 | 23 | 4 | 19 |
| Nov 2023 | 15 | 2 | 13 | 15 | 2 | 13 |
| Dec 2023 | 13 | 1 | 12 | 13 | 1 | 12 |
| Jan 2024 | 5 | 0 | 5 | 5 | 0 | 5 |
| Feb 2024 | 6 | 0 | 6 | 6 | 0 | 6 |
| Mar 2024 | 10 | 0 | 10 | 10 | 0 | 10 |
| Apr 2024 | 11 | 2 | 9 | 11 | 2 | 9 |
| May 2024 | 6 | 1 | 5 | 6 | 1 | 5 |
| Jun 2024 | 5 | 0 | 5 | 5 | 0 | 5 |
| Jul 2024 | 7 | 0 | 7 | 7 | 0 | 7 |
| Aug 2024 | 6 | 0 | 6 | 6 | 0 | 6 |
| Sep 2024 | 4 | 0 | 4 | 4 | 0 | 4 |
| **Total** | **395** | **211** | **184** | **238** | **54** | **184** |

^†^ Questions on the impact of mpox on sexual and drug use behaviours were included from November 2022 onwards.

Abbreviations: MSM, Men who have sex with men

**
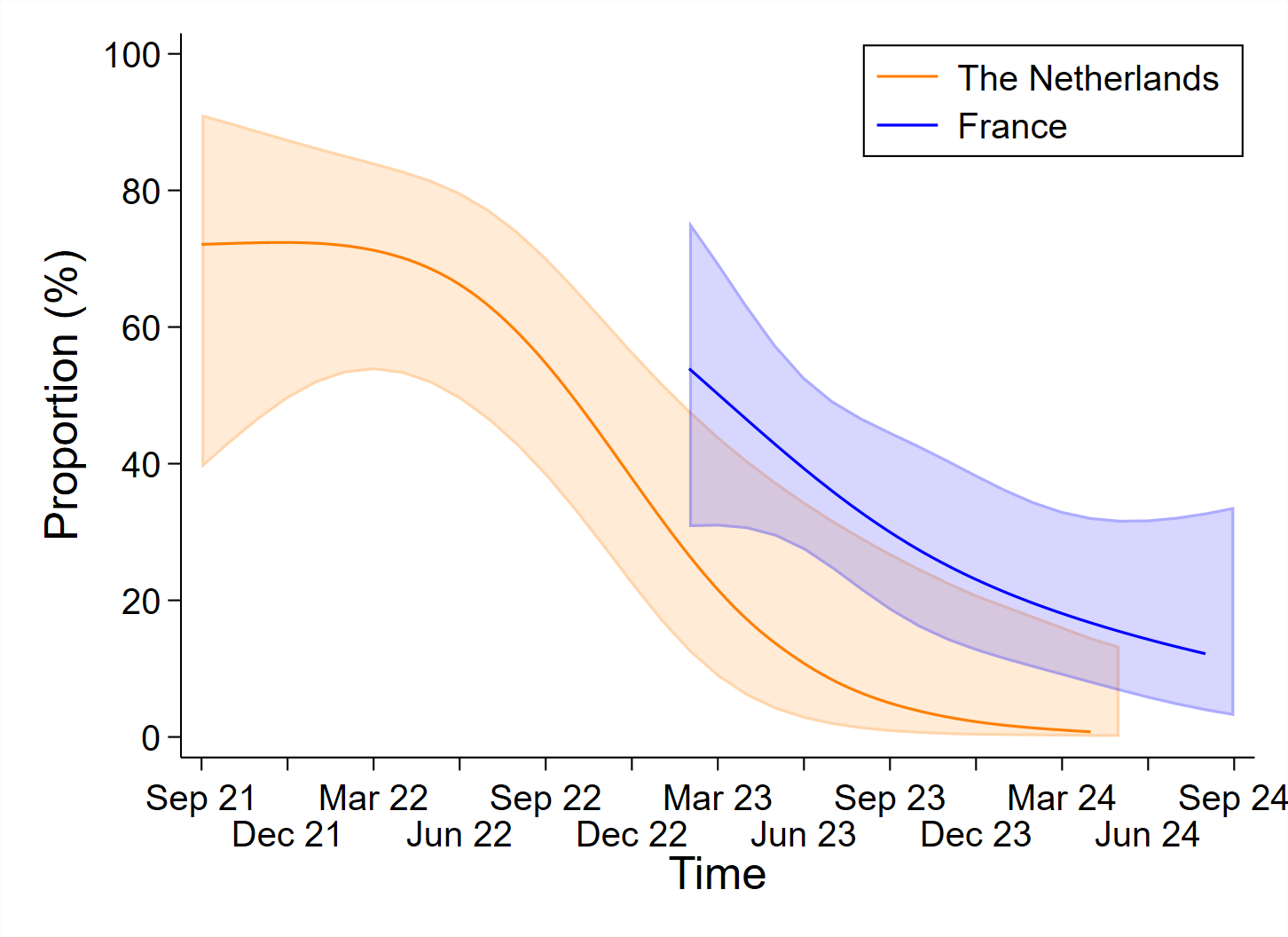
**

# **Supplementary Figure 3.** Proportion reporting any effect of COVID-19 restrictions on sexual and drug use behaviour associated with HCV in the preceding six months over calendar time stratified by country of enrolment among included MSM between September 2021 and September 2024.

Fitted solid line represents the predicted proportions per calendar month. The shaded area surrounding each line represents the 95% confidence interval.

Abbreviations: HCV, Hepatitis C virus; MSM, Men who have sex with men

# **Supplementary Table 2.** Determinants of reporting any effect of the COVID-19 restrictions among MSM enrolled in the ICECREAM study between September 2021 and September 2024.

|  | | **Univariable analysis** | | |  | **Multivariable analysis** | | | |
| --- | --- | --- | --- | --- | --- | --- | --- | --- | --- |
|  | | **(n=220)** | | |  | **(n=208)** | | | |
|  | | **OR**^†^ | **95% CI** | ***p*** |  | **aOR**^†^ | | **95% CI** | ***p*** |
| **Age (per 10 years)**^‡^ | | 1.44 | 0.98-2.12 | 0.07 |  | 1.59 | | 1.04-2.44 | 0.03 |
| **Educational level**^‡^ | |  |  |  |  |  | |  |  |
|  | ≤High school degree | Ref |  |  |  | Ref |  | |  |
|  | >High school degree | 1.46 | 0.59-3.61 | 0.41 |  | 1.30 | 0.53-3.23 | | 0.57 |
| **HIV status** | |  |  |  |  |  | |  |  |
|  | Negative | Ref |  |  |  | Ref |  | |  |
|  | Positive | 0.92 | 0.36-2.34 | 0.86 |  | 0.53 | 0.19-1.48 | | 0.23 |
| **Number of HCV infections**^‡^ | |  |  |  |  |  | |  |  |
|  | 1 | Ref |  |  |  | Ref |  | |  |
|  | ≥2 | 1.32 | 0.53-3.32 | 0.55 |  | 1.40 | 0.56-3.50 | | 0.47 |
| **Group sex / chemsex**^§^ | | 1.39 | 0.63-3.10 | 0.42 |  | 0.99 | | 0.40-2.44 | 0.98 |
| **HCV-MOSAIC risk score (continuous)** | | 1.25 | 1.00-1.57 | 0.05 |  | 1.20 | | 0.94-1.53 | 0.14 |

^†^ Indicated the relative increase or decrease in odds when present versus absent at study visits.

^‡^ 12 participants with missing data.

^§^ Defined as the use of methamphetamine, γ-hydroxybutyric acid (GHB)/γ-butyrolactone (GBL) and/or mephedrone before or during sex.

Abbreviations: aOR, Adjusted odds ratio; CAS, Condomless anal sex; CI, Confidence interval; HCV, Hepatitis C virus; IDU, Injecting drug use; MOSAIC, MSM Observation Study of Acute Infection with HCV; OR, Odds ratio; MSM, Men who have sex with men; Ref, Reference category; STI, Sexually transmitted infection; 6M, during the previous six months

# **Supplementary Table 3.** Comparison of characteristics at baseline of MSM who are included vs. excluded in the analyses of the impact of mpox measures on sexual and drug use behaviours between 2021-2024.

|  |  | **Included (n=137)^†^** | |  | **Excluded (n=71)^†^** | |  | ***p*** |
| --- | --- | --- | --- | --- | --- | --- | --- | --- |
|  |  | **n^‡^** | **%^‡^** |  | **n^‡^** | **%^‡^** |  |  |
| *Sociodemographic characteristics* | | | | | | | | |
| **Age, years** | |  |  |  |  |  |  |  |
|  | Median [IQR] | 52 | [45-58] |  | 51 | [44-58] |  | 0.74 |
|  | ≤34 | 8 | 5.8% |  | 4 | 5.6% |  | 0.99 |
|  | 35-44 | 26 | 19.0% |  | 14 | 19.7% |  |  |
|  | ≥35 | 103 | 75.2% |  | 53 | 74.7% |  |  |
| **Country of enrolment** | |  |  |  |  |  |  | <0.001 |
|  | The Netherlands | 40 | 29.2% |  | 71 | 100.0% |  |  |
|  | France | 97 | 70.8% |  | 0 | 0.0% |  |  |
| **Higher than high school degree** | | 104 | 75.9% |  | 57 | 80.3% |  | 0.48 |
| *HIV and HCV clinical characteristics* | | | | | | | | |
| **HIV status and PrEP use** | |  |  |  |  |  |  | 0.52 |
|  | Positive | 115 | 83.9% |  | 56 | 78.9% |  |  |
|  | Negative and using PrEP | 19 | 13.9% |  | 14 | 19.7% |  |  |
|  | Negative and not using PrEP | 3 | 2.2% |  | 1 | 1.4% |  |  |
| **Years since HIV diagnosis**^§^ | |  |  |  |  |  |  |  |
|  | Median [IQR] | 18.3 | [12.8-24.3] |  | 16.6 | [12.6-22.7] |  | 0.56 |
| **On ART**^§^ | | 115 | 100.0% |  | 56 | 100.0% |  | 1.00 |
| **Number of previous HCV infections** | |  |  |  |  |  |  | 0.80 |
|  | 1 | 104 | 75.9% |  | 55 | 77.5% |  |  |
|  | ≥2 | 33 | 24.1% |  | 16 | 22.5% |  |  |
| **Years since last HCV diagnosis** | |  |  |  |  |  |  |  |
|  | Median [IQR] | 5.2 | [1.9-9.8] |  | 4.8 | [2.8-9.2] |  | 0.75 |
| **Treatment of last HCV infection** | |  |  |  |  |  |  | 0.58 |
|  | Pegylated interferon based | 51 | 37.2% |  | 30 | 42.3% |  |  |
|  | DAA based | 72 | 52.6% |  | 32 | 45.1% |  |  |
|  | No treatment (spontaneous clearance) | 14 | 10.2% |  | 9 | 12.7% |  |  |
| *Sexual and drug use behaviours^6M^* | | | | | | | | |
| **Stable sexual partner** | | 62 | 45.3% |  | 31 | 43.7% |  | 0.83 |
| **Casual partner** | | 113 | 82.5% |  | 59 | 83.1% |  | 0.91 |
| **Number of casual partners** | |  |  |  |  |  |  |  |
|  | Median [IQR] | 10 | [3-30] |  | 10 | [3-30] |  | 0.76 |
| **Receptive CAS** | | 114 | 83.2% |  | 59 | 83.1% |  | 0.98 |
| **Unprotected fisting** | | 54 | 39.4% |  | 28 | 39.4% |  | 0.99 |
| **Sharing of sex toys** | | 35 | 25.6% |  | 23 | 32.4% |  | 0.30 |
| **Sharing of straws** | | 44 | 32.1% |  | 26 | 36.6% |  | 0.52 |
| **IDU** | | 24 | 17.5% |  | 13 | 18.3% |  | 0.89 |
| **Any STI** | | 55 | 40.2% |  | 24 | 33.8% |  | 0.37 |
| **Ulcerative STI**^¶^ | | 37 | 27.0% |  | 12 | 16.9% |  | 0.10 |
| **SDU**^††^ | | 92 | 67.2% |  | 47 | 66.2% |  | 0.89 |
| **Chemsex**^‡‡^ | | 76 | 55.5% |  | 38 | 53.5% |  | 0.79 |
| **Group sex** | | 89 | 65.0% |  | 29 | 40.9% |  | 0.001 |
| **HCV-MOSAIC risk score [9, 10]** | |  |  |  |  |  |  |  |
|  | Median [IQR] | 2.3 | [1.1-3.5] |  | 2.1 | [1.1-3.5] |  | 0.94 |

P-value represents the statistical comparison between country of enrolment using Pearson’s χ^2^ or Fisher’s exact test for categorical variables and Student’s t-test or Mann-Whitney U test for continuous variables.

^†^ 9 participants who were included in and 3 participants who were excluded from the mpox analysis did not complete the baseline questionnaire and were excluded from the analysis.

^‡^ Unless otherwise indicated.

^§^ Only among those with an HIV infection.

^¶^ Having a syphilis, herpes or Lymphogranuloma Venereum infection at study visit.

^††^ Defined as the use of any drugs before or during sex, excluding alcohol.

^‡‡^ Defined as the use of methamphetamine, γ-hydroxybutyric acid (GHB)/γ-butyrolactone (GBL) and/or mephedrone before or during sex.

Abbreviations: ART, Antiretroviral therapy; CAS, Condomless anal sex; DAA, Direct acting antiviral; HCV, Hepatitis C virus; HIV, Human immunodeficiency virus; ICECREAM, Interventions to curb hepatitis C reinfection among MSM; IDU, Injecting drug use; IQR, Interquartile range; MOSAIC, MSM Observational Study of Acute Infection with HCV; MSM, Men who have sex with men; PrEP, Pre-exposure prophylaxis; SDU, sexualized drug use; STI, Sexually transmitted infection; 6M, in the previous 6 months.

# **Supplementary Table 4.** Determinants of reporting any effect of the mpox outbreak among MSM enrolled in the ICECREAM study between November 2022 and September 2024.

|  | | **Univariable analysis** | | |  | **Multivariable analysis** | | |
| --- | --- | --- | --- | --- | --- | --- | --- | --- |
|  | | **(n=146)** | | |  | **(n=137)** | | |
|  | | **OR**^†^ | **95% CI** | ***p*** |  | **aOR**^†^ | **95% CI** | ***p*** |
| **Age (per 10 years)**^‡^ | | 1.02 | 0.72-1.44 | 0.90 |  | 1.16 | 0.78-1.71 | 0.46 |
| **Educational level**^‡^ | |  |  |  |  |  |  |  |
|  | ≤High school degree | Ref |  |  |  | Ref |  |  |
|  | >High school degree | 1.97 | 0.79-4.92 | 0.15 |  | 1.77 | 0.68-4.62 | 0.24 |
| **HIV status** | |  |  |  |  |  |  |  |
|  | Negative | Ref |  |  |  | Ref |  |  |
|  | Positive | 0.41 | 0.16-1.06 | 0.07 |  | 0.35 | 0.12-1.05 | 0.06 |
| **Number of HCV infections**^‡^ | |  |  |  |  |  |  |  |
|  | 1 | Ref |  |  |  | Ref |  |  |
|  | ≥2 | 1.94 | 0.80-4.67 | 0.14 |  | 1.87 | 0.74-4.72 | 0.18 |
| **Group sex / chemsex**^§^ | | 0.99 | 0.42-2.33 | 0.97 |  | 1.12 | 0.43-2.91 | 0.81 |
| **HCV-MOSAIC risk score (continuous)** | | 0.94 | 0.76-1.18 | 0.61 |  | 0.87 | 0.67-1.15 | 0.33 |

^†^ Indicated the relative increase or decrease in odds when present versus absent at study visit.

^‡^ 9 participants with missing data.

^§^ Defined as the use of methamphetamine, GHB/GBL and/or mephedrone before or during sex.

Abbreviations: aOR, Adjusted odds ratio; CAS, Condomless anal sex; CI, Confidence interval; HCV, Hepatitis C virus; IDU, Injecting drug use; ; MOSAIC, MSM Observation Study of Acute Infection with HCV; OR, Odds ratio; MSM, Men who have sex with men; Ref, Reference category; STI, Sexually transmitted infection; 6M, during the previous six months

**
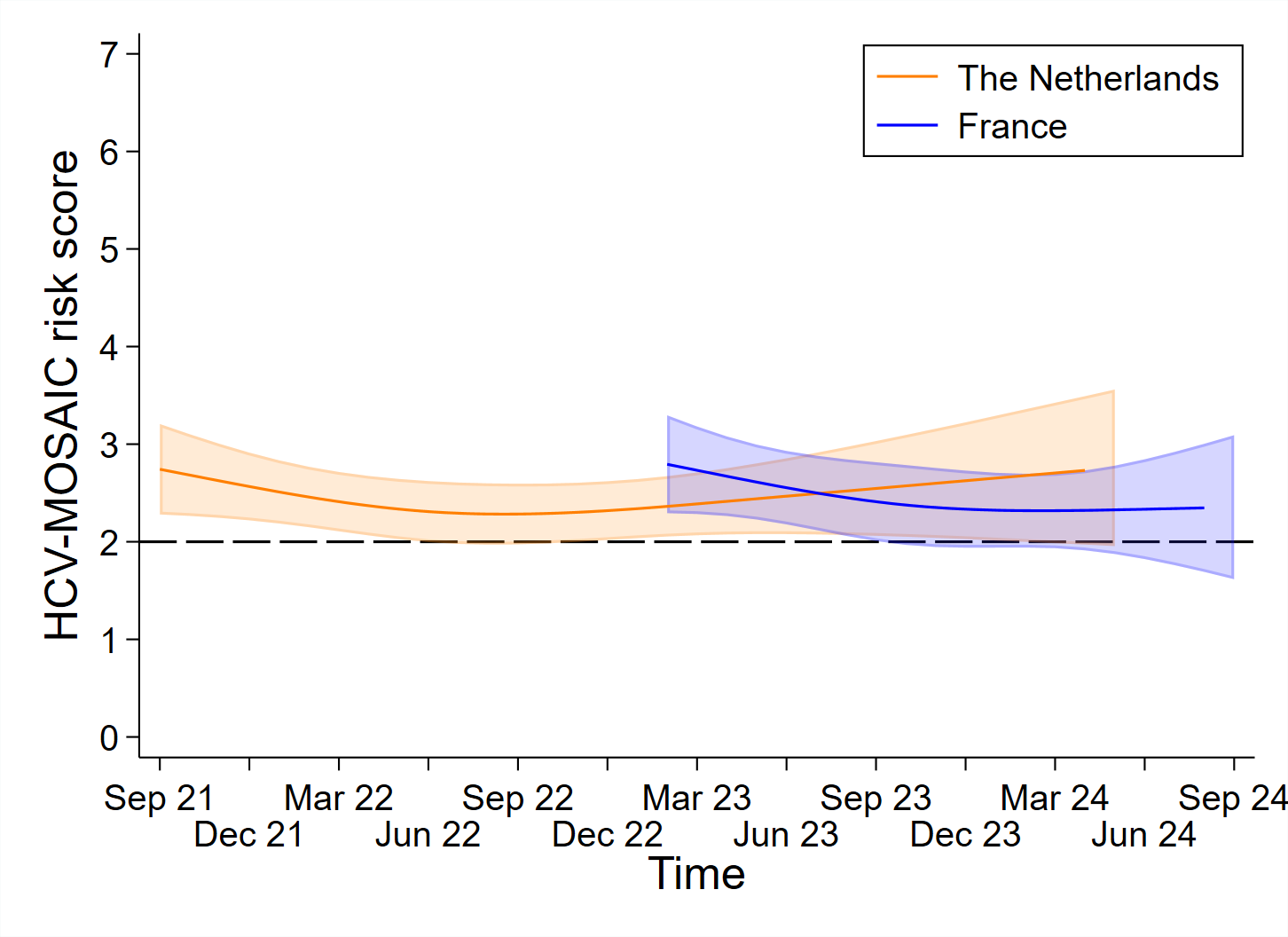
**

# **Supplementary Figure 4**. Average predicted HCV-MOSAIC risk score over time stratified by country of enrolment among included MSM between September 2021 and September 2024.

Fitted solid line represent the average predicted HCV-MOSAIC risk per calendar month. The shaded area surrounding each line represent the 95% confidence interval. The dashed black line represents the HCV-MOSAIC risk score cut-off, with a risk score ≥2.0 indicating high risk of HCV infection.

Abbreviations: HCV, Hepatitis C virus; MOSAIC, MSM Observational Study of Acute Infection with hepatitis C; MSM, Men who have sex with men

**
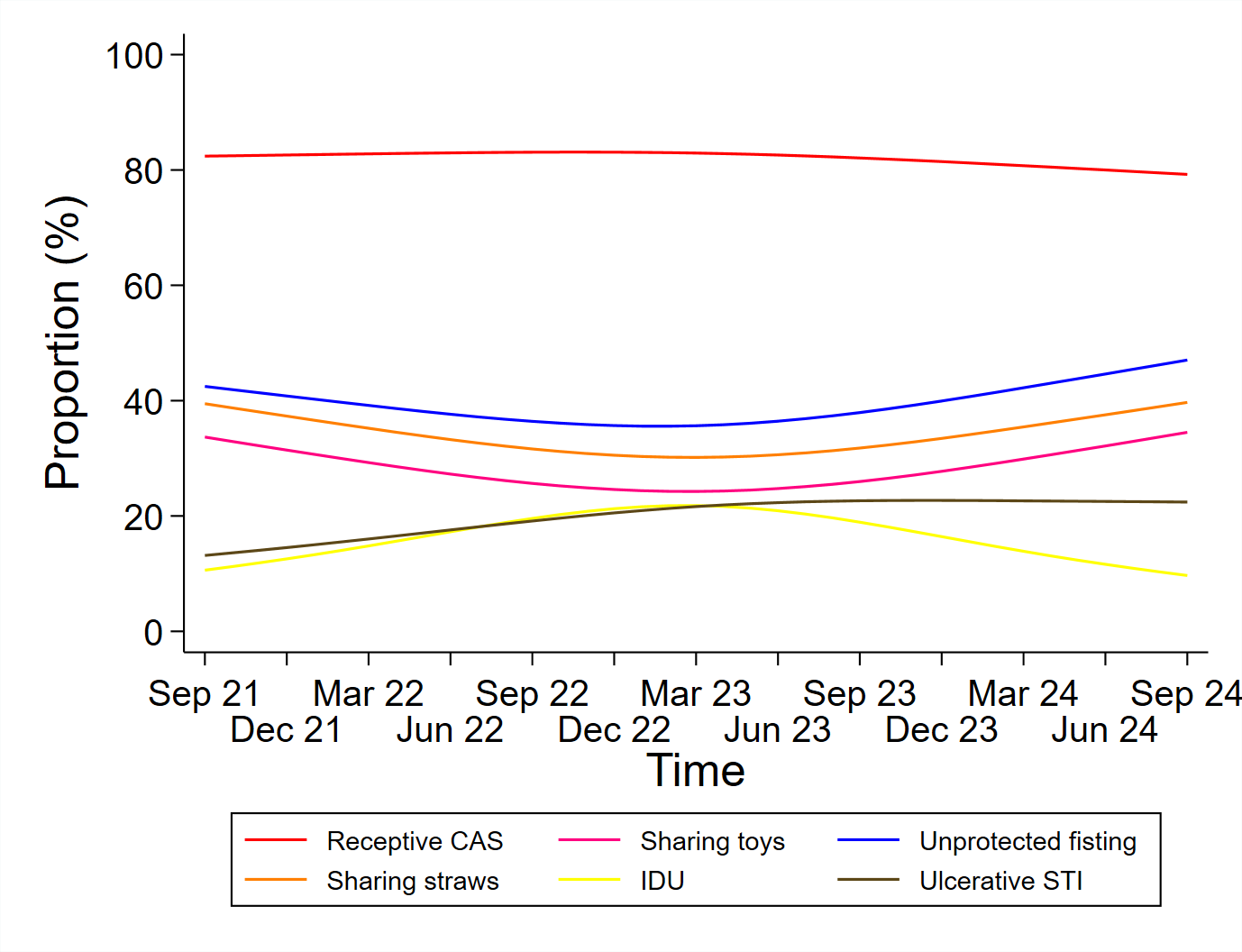
**

# **Supplementary Figure 5.** Predicted proportion reporting individual risk behaviours included in the HCV-MOSAIC risk score in the preceding six months over calendar time among included MSM between September 2021 and September 2024 [9, 10].

Abbreviations: CAS, Condomless anal sex; HCV, hepatitis C virus; IDU, Injecting drug use; MOSAIC; MSM Observational Study of Acute Infection with hepatitis C; MSM, Men who have sex with men; STI, Sexually transmitted infection

# **References**

1. Roser M. What is the COVID-19 Stringency Index? Available from: <https://ourworldindata.org/metrics-explained-covid19-stringency-index>: Accessed on: 6 December 2024; 2021.

2. National Institute for Public Health and the Environment. Variants of the coronavirus SARS-CoV-2 [in Dutch]. Available at: <https://www.rivm.nl/en/coronavirus-covid-19/current/variants> Accessed September 23, 2024.

3. Maisa A, Spaccaferri G, Fournier L, Schaeffer J, Deniau J, Rolland P, et al. First cases of Omicron in France are exhibiting mild symptoms, November 2021-January 2022. Infect Dis Now. 2022;52(3):160-4.

4. Rijksoverheid. Coronavirus tijdlijn. Available at: <https://www.rijksoverheid.nl/onderwerpen/coronavirus-tijdlijn>. Accessed September 23, 2024.

5. BBC. Covid: France tightens restrictions amid Omicron surge. Published 28 December 2021: Available at: <https://www.bbc.com/news/world-europe-59805829>.

6. Mathieu E SF, Dattani S, Ritchie H, Roser M,. Mpox. Available at: <https://ourworldindata.org/mpox>. Accessed September 29, 2024.

7. van Ewijk CE, Miura F, van Rijckevorsel G, de Vries HJ, Welkers MR, van den Berg OE, et al. Mpox outbreak in the Netherlands, 2022: public health response, characteristics of the first 1,000 cases and protection of the first-generation smallpox vaccine. Euro Surveill. 2023;28(12).

8. Lepelletier D, Pozzetto B, Chauvin F, Chidiac C, High Council for Public Health national working g. Management of patients with monkeypox virus infection and contacts in the community and in healthcare settings: a French position paper. Clin Microbiol Infect. 2022;28(12):1572-7.

9. Newsum AM, Stolte IG, van der Meer JT, Schinkel J, van der Valk M, Vanhommerig JW, et al. Development and validation of the HCV-MOSAIC risk score to assist testing for acute hepatitis C virus (HCV) infection in HIV-infected men who have sex with men (MSM). Euro Surveill. 2017;22(21):30540.

10. Hage K, van de Kerkhof M, Boyd A, Carson JM, Newsum AM, Matser A, et al. Screening for Hepatitis C Virus Reinfection Using a Behaviour-Based Risk Score among Men Who Have Sex with Men with HIV: Results from a Case-Control Diagnostic Validation Study. Pathogens. 2023;12(10):1248.
